# Molecular characterization of novel sulfotransferases from the tick, *Ixodes scapularis*

**DOI:** 10.1186/1471-2091-12-32

**Published:** 2011-06-27

**Authors:** Sivakamasundari Pichu, Emine B Yalcin, José MC Ribeiro, Roberta S King, Thomas N Mather

**Affiliations:** 1 Center for Vector-Borne Disease, University of Rhode Island, Kingston, RI 02881, USA; 2 Department of Biomedical and Pharmaceutical Sciences, College of Pharmacy, University of Rhode Island, Kingston, RI, 02881, USA; 3 Section of Vector Biology, Laboratory of Malaria and Vector Research, National Institute of Allergy and Infectious Diseases, National Institutes of Health, Bethesda, Maryland, 20892, USA

## Abstract

**Background:**

*Ixodes scapularis*, commonly known as the blacklegged or deer tick, is the main vector of Lyme disease in the United States. Recent progress in transcriptome research has uncovered hundreds of different proteins expressed in the salivary glands of hard ticks, the majority of which have no known function, and include many novel protein families. We recently identified transcripts coding for two putative cytosolic sulfotransferases in these ticks which recognized phenolic monoamines as their substrates. In this current study, we characterize the genetic expression of these two cytosolic sulfotransferases throughout the tick life cycle as well as the enzymatic properties of the corresponding recombinant proteins. Interestingly, the resultant recombinant proteins showed sulfotransferase activity against both neurotransmitters dopamine and octopamine.

**Results:**

The two sulfotransferase genes were coded as *Ixosc *SULT 1 & 2 and corresponding proteins were referred as *Ixosc *Sult 1 and 2. Using gene-specific primers, the sulfotransferase transcripts were detected throughout the blacklegged tick life cycle, including eggs, larvae, nymphs, adult salivary glands and adult midgut. Notably, the mRNA and protein levels were altered upon feeding during both the larval and nymphal life stages. Quantitative PCR results confirm that *Ixosc *SULT1 was statistically increased upon blood feeding while *Ixosc *SULT 2 was decreased. This altered expression led us to further characterize the function of these proteins in the Ixodid tick. The sulfotransferase genes were cloned and expressed in a bacterial expression system, and purified recombinant proteins *Ixosc *Sult 1(R) and 2(R) showed sulfotransferase activity against neurotransmitters dopamine and octopamine as well as the common sulfotransferase substrate *p-*nitrophenol. Thus, dopamine- or octopamine-sulfonation may be involved in altering the biological signal for salivary secretion in *I. scapularis.*

**Conclusions:**

Collectively, these results suggest that a function of *Ixosc *Sult 1 and Sult 2 in *Ixodid *tick salivary glands may include inactivation of the salivation signal via sulfonation of dopamine or octopamine.

## Background

Ticks are hematophagous arthropods, notorious as vectors of human and animal pathogens [1]. Diseases transmitted by ticks are global medical and veterinary public health problems [2]. *Ixodes scapularis*, known commonly as the deer tick or blacklegged tick, is the major vector for Lyme disease, babesiosis, and granulocytic anaplasmosis to humans and domestic animals in the United States. Ticks and other blood-feeding arthropod vectors manipulate host hemostatic and immune responses by secreting molecules from their multifunctional salivary glands. Blood feeding by ticks requires prolonged contact with host tissues and blood, and it has been suggested that the adaptation of ticks to their natural host has resulted in evolution of an appropriate set of salivary components allowing the tick to evade host immunity and prevent coagulation at the feeding site to successfully obtain its blood meal [3]. The blood feeding cycle of larval and nymphal *Ixodes scapularis *typically extends for three to four days, while that of the adult female blacklegged tick lasts approximately six days; during this time the tick alternately secretes salivary fluid into the host and takes up blood from the host [4]. Successful blood feeding must require endogenous signalling molecules within the tick to turn on and off salivation and control the release of some 300 secreted salivary proteins into the saliva [5]. One possible strategy for reducing tick-transmitted disease incidence could involve manipulating tick salivary secretion so as to interrupt or shorten the duration of host attachment or blood feeding. Accordingly, this research is focussed on elucidating biochemical pathways of two sulfotransferase genes that may be involved in tick salivation and pathogen transmission.

Sulfotransferases catalyze transfer of a sulfonyl moiety (-SO_3_) from the universal donor 3'-phosphoadenosine-5'-phosphosulfate (PAPS) to an oxygen or nitrogen acceptor, resulting in production of a sulfate ester. Historically, sulfotransferases have been classified by their sub-cellular localization as either cytosolic or membrane bound [6]. In general, cytosolic sulfotransferases accept relatively small molecules as substrates, while the membrane bound sulfotransferases utilize macromolecules [6-9].

Although a number of sulfotransferase genes are registered in various invertebrate genome databases, only a few invertebrate sulfotransferases have been characterized. Cytosolic sulfotransferases have been characterized from the nematode *Caenorhabditis elegans *[10] and from the arthropods *Bombyx mori *[11], *Spodoptera frugiperda *[12], and *Drosophila melanogaster *[13,14]. *C. elegans *contains a glycosaminoglycan membrane bound sulfotransferase with heparan-2-*O-*sulfotransferase activity [15]. Membrane bound tyrosylprotein sulfotransferases have been characterized from *Drosophila melanogaster *and from *C. elegans *[16]. Specifically in salivary glands, tyrosylprotein sulfotransferases have been characterized from human saliva [17] and rat salivary gland tissue [16,18-20]. Thus, precedent exists for both cytosolic and membrane-localized sulfotransferases in arthropods, and in mammalian salivary glands.

Sulfotransferase enzymes modulate the activity of many hormones and proteins; in humans and other mammalian species, some are involved in regulating immune responses and blood coagulation [6,9,21-25]. Recently, we predicted that dopamine and octopamine could serve as substrates for tick-derived sulfotransferases [26]. Dopamine is known to stimulate secretion of tick saliva through a neurochemical mechanism. Tick salivary fluid secretion is controlled via a dopamine D1 receptor and cAMP dependent protein phosphorylation cascade following salivary gland stimulation by dopamine released from nerve endings [27]. Moreover, endogenous dopamine has been identified in salivary glands of the ticks *Rhipicephalus microplus *and *Amblyomma hebraeum *[28,29]. Octopamine also exhibits neuromodulatory functions in arthropods [30]. Thus, precedent exists for sulfotransferase enzymes to impact host hemostasis as well as modulate tick salivary or host components which regulate salivation, immune response, or blood coagulation.

Our recent report showed the three dimensional structural conservation of two novel tick sulfotransferases (*Ixosc *Sult 1 and Sult 2) and its ligand docking. Predictions from the modelling were tested and confirmed using native tissue enzyme homogenates from larval and nymphal stage blacklegged ticks [26]. In this present study, we identify the genetic expression of two cytosolic sulfotransferases throughout the tick life cycle and characterize the expressed recombinant proteins. Interestingly, we observed differential gene expression between the two sulfotransferases during blood feeding, and that their recombinant enzymes effectively sulfate neurotransmitters dopamine and octopamine. Taken together, the observed differential expression of these two sulfotransferase genes and the resulting protein affinity for neurotransmitters involved in tick salivation suggests that *Ixosc *Sult 1 and Sult 2 may play roles in tick salivation and pathogen transmission processes.

## Results

### Amino acid sequence comparison

**Figure 1:**
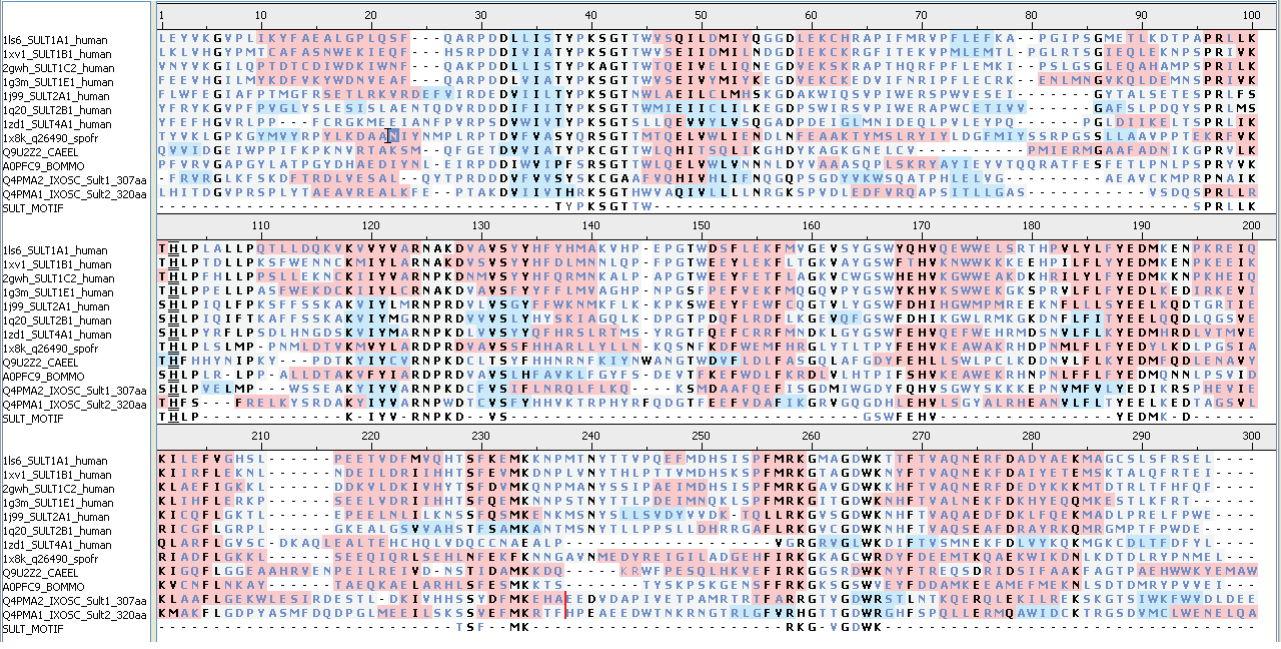
**Sequence Alignment of *Ixosc Sult 1 *and *2 *with selected known cytosolic sulfotransferases**. *Ixosc Sult 1 *and *2 *are designated as q4pma2_*Ixosc*_Sult1_307aa and q4pma1_*Ixosc*_Sult2_320aa, respectively. Human sulfotransferases are listed with a protein data bank (PDB) code for a matching x-ray crystal structure. *C. elegans *and *B. mori *sulfotransferases are listed with their UniProt code. SULT_MOTIF indicates the residues which make up the cytosolic sulfotransferase amino acid sequence motif regions.

The sequence alignment of the two *Ixodes *sulfotransferases (*Ixosc *Sult 1 and 2) and related mammalian cytosolic sulfotransferases are shown in Figure 1. The SULT_MOTIF on Figure 1 indicates the residues which make up the cytosolic sulfotransferase amino acid sequence motif regions. Thus, the amino acid sequence comparison confirms that there is presence of conserved domains of cytosolic sulfotransferases on both *Ixosc *Sult 1 and 2.

### Transcriptional expression of *Ixosc *SULT 1 and SULT 2 genes and gene products

**Figure 2:**
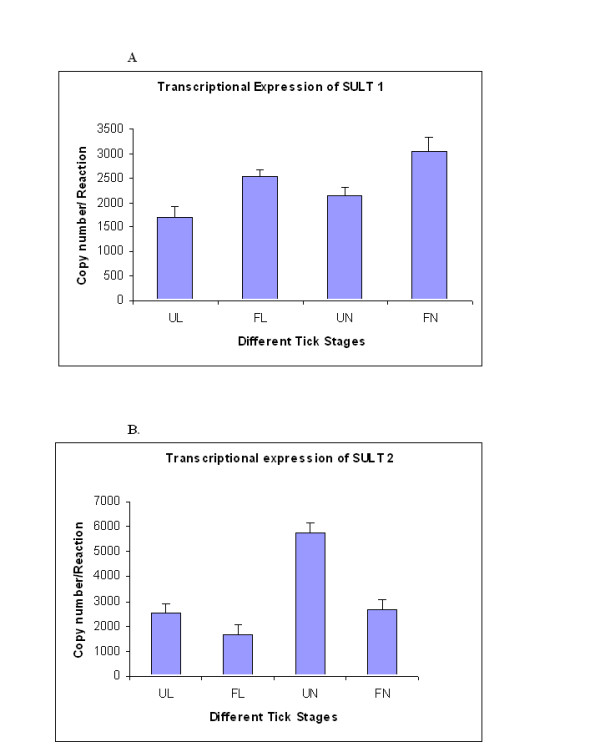
**Quantitative analysis of *Ixosc SULT 1 and SULT 2 *mRNA Expression**. Total RNA was extracted from different developmental stages and PCR amplified with gene specific primers. Quantitative transcriptional expression was determined as described in Materials and Methods. The PCR rates of *SULT 1 *(A) and *SULT 2 *(B), in various stages of the tick life cycle were normalized to the rate of synthesis of β-actin, included as the endogenous control. Data were plotted as copy numbers per reaction to samples at various level of feeding. Bars indicate PCR values as the Mean ± SD of three replicated experiments. Abbreviations: UL - Unfed Larvae, FL - Fed Larvae, UN - Unfed Nymph, FN - Fed Nymph.

To shed light on the transcriptional control of the two genes during different life stages of *I. scapularis*, we used real-time quantitative RT-PCR to amplify RNA isolated from pooled unfed and blood-fed whole larvae and nymphs, as well as the salivary glands and midguts dissected from both unfed and blood-fed adult stage ticks. Usually, the feeding time varied between 48 to 72 hours. Expression levels were normalized using the constitutively expressed β-actin transcript as a standard. Results showed that the *SULT 1 *was increased during feeding 1.5 fold in larvae and 1.4 fold in nymphs. In contrast, *SULT 2 *was decreased during feeding by 1.5 fold in larvae and 2.6 fold in nymphs as shown in Figure 2A and 2B. Furthermore, the difference in transcript abundance for *SULT 1 *and *SULT 2 *were statistically significant, p < 0.03 for *SULT 1 *and p < 0.01 for *SULT 2 *during larval stages between unfed and fed, where as p < 0.008 and p < 0.03 for *SULT 2 *during nymphal stages between unfed and fed, respectively (data not shown). Briefly, as feeding initiated in larval ticks, *SULT 1 *expression increased whereas *SULT 2 *expression decreased during feeding in both larval and nymphal stages. During the adult tick feeding, there was no significant difference in *SULT 1 *or *SULT 2 *transcript abundance (unfed salivary gland: 3120 ± 310; fed salivary gland: 3510 ± 260; unfed midgut: 3350 ± 320; fed midgut: 3620 ± 310, copy number/reaction respectively).

**Figure 3:**
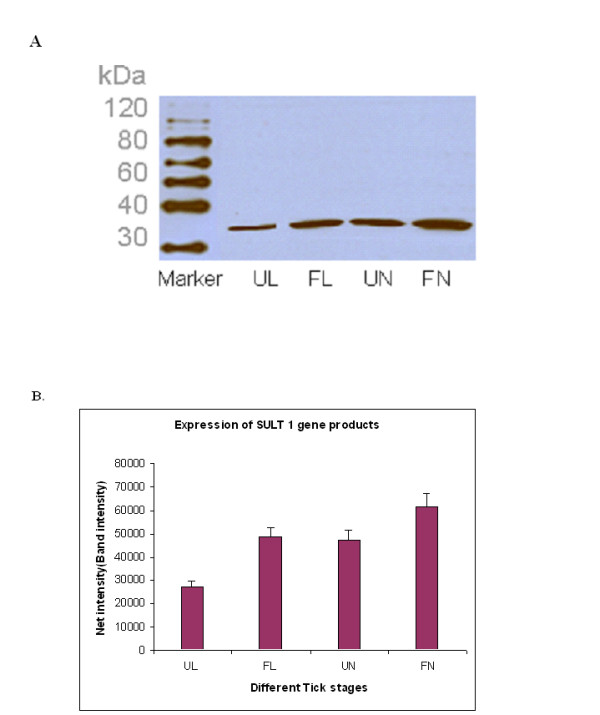
**Analysis of *Ixosc *Sult 1 protein expression during feeding**. (A) *Ixosc *Sult 1 detected by Western blot, (B) Quantitation of band intensity. Tissue homogenates from different developmental stages were analyzed by Western blot using anti-Sult 1 (R) raised antibody. Lanes: 1. Magic Marker, 2. Unfed larvae, 3. Fed larvae, 4. Unfed nymph, 5. Fed nymph. *SULT 1 *gene products were quantified using Kodak Digital Science 1D image analysis software and the net intensity/band intensity was plotted against the different tick stages.

**Figure 4:**
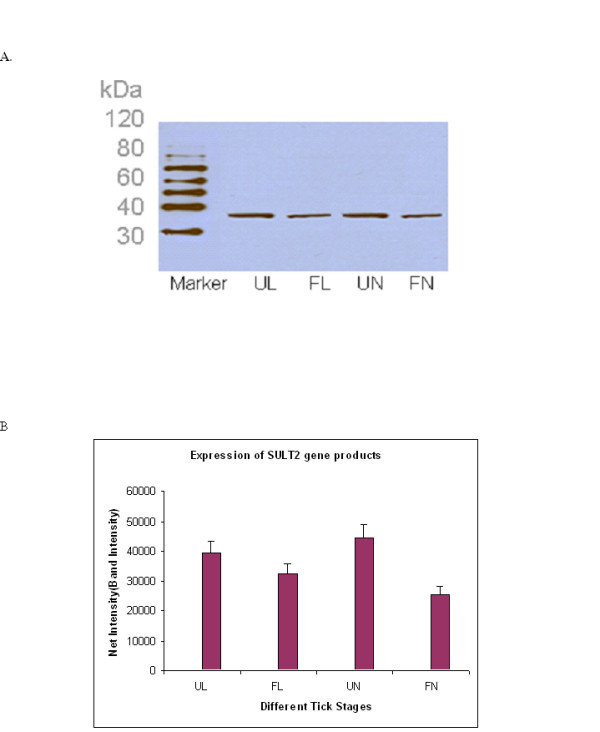
**Analysis of *Ixosc *Sult 2 protein expression during feeding**. (A) *Ixosc *Sult 2 detected by Western blot, (B) Quantitation of band intensity. Tissue homogenates from different developmental stages were analyzed by Western blot using anti-Sult 2 (R) raised antibody. Lanes: 1. Magic Marker, 2. Unfed larvae, 3. Fed larvae, 4. Unfed nymph, 5. Fed nymph. *SULT 2 *gene products were quantified using Kodak Digital Science 1D image analysis software and the net intensity/band intensity was plotted against the different tick stages.

Similar results were observed at the protein level using Western blot probed with antibodies raised against expressed and affinity-purified *Ixosc *Sult 1 or Sult 2. Band intensities were quantified using Kodak Digital Science 1D Image Analysis Software and plotted against different tick stages (Figure 3A and 3B; Figure 4A and 4B). Values are expressed as mean ± SD for three replicated experiments and show significant differences between blood-fed and unfed states, respectively, at both the nymphal and larval stages. Similar to the quantitative PCR results, the *Ixosc *Sult 1 and 2 protein levels in the adult stage showed no difference on feeding (data not shown).

**Figure 5:**
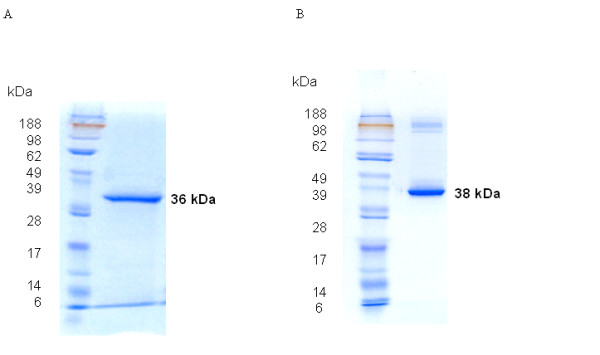
**SDS-PAGE of expressed *Ixosc *Sult 1 (R) (A), Sult 2 (R)(B)**. The expressed purified recombinant proteins were analyzed and stained with Coomassie brilliant blue. Lanes: 1. Prestained molecular weight marker, 2. Purified protein.

Full length *Ixosc SULT 1 *and *SULT 2 *were cloned into the pTrcHis2 TOPO TA expression vector. The expressed proteins were purified using a HIS-Select ILAP (Sigma) column. The purified *Ixosc *Sult 1(R) and Sult 2(R) showed molecular weights of 36 and 38 kDa, respectively (Figure 5A &5B), and the purified proteins were sequenced using liquid automatic sequencing. The resulting sequences from the Expert Protein Analysis System (ExPASy) confirmed that the expressed proteins are from tick sulfotransferase genes (data not shown).

### Enzymatic characterization of *Ixosc *Sult 1 (R) and Sult 2 (R)

**Figure 6:**
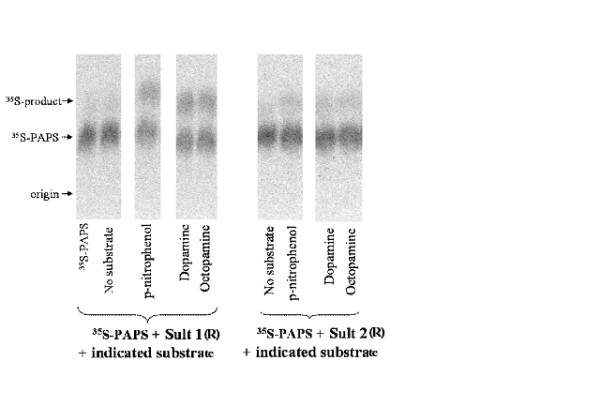
**Dopamine, octopamine, and *p-*nitrophenol as substrates for *Ixosc *Sult 1 (R) and Sult 2 (R)**. TLC separation of ^35^S-labeled product from PAP^35^S (4 μM) after incubation of 10 μM of noted compounds with expressed purified *Ixosc *Sult 1 (R) or Sult 2 (R)as described in Experimental Procedures.

**Figure 7:**
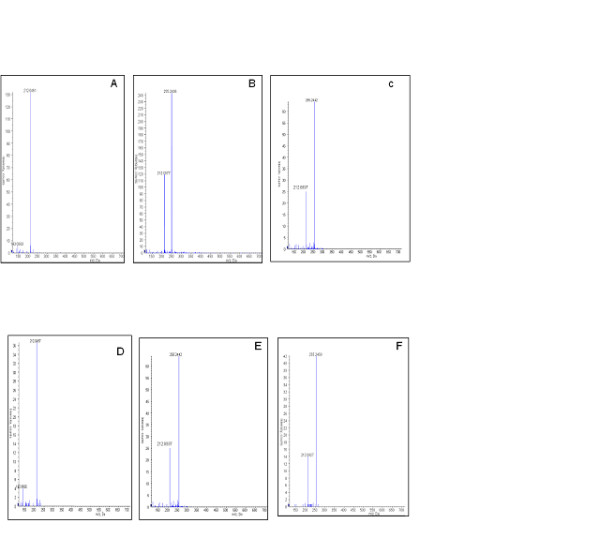
**Mass Spectra of dopamine-sulfate and octopamine-sulfate formed by incubating *Ixosc *Sult 1(R) or *Ixosc *Sult 2 (R)**. **A**: Sult 1 (R) with PAP^35^S (Control), **B**: Sult 1(R) + PAP^35^S + Dopamine, **C**: Sult 1 (R) + PAP^35^S + Octopamine, **D**: Sult 2 (R) + PAP^35^S (Control), **E**: Sult 2 (R) + PAP^35^S + Dopamine, **F**: Sult 2 (R) + PAP^35^S + Octopamine.

We tested whether expressed purified *Ixosc *Sult 1 (R) or Sult 2 (R) could sulfonate selected potentially relevant substrates. The rationale for including p-nitrophenol, 17b-estradiol, pregnenolone, and dopamine was that they are prototype substrates for the mammalian sulfotransferases. The rationale for including octopamine and an additional rationale for including dopamine was based on the relevance of these compounds as arthropod neuromodulators and on the previous structure modelling study [26] which predicted these monoamines as potential substrates for the *I. scapularis *sulfotransferases. Arthropod steroids such as ecdysone were not tested as substrates, as the previous structure modelling study indicated that these steroid structures are not complimentary to the binding pocket of the *I. scapularis *sulfotransferases and should not be substrates [26]. Each potential substrate was added to 4 μM PAP^35^S (in water) and purified enzyme (0.03-0.04 mg/mL in 20 mM potassium phosphate pH 7.0), and was incubated at 37°C. Because of the zwitterionic nature of two of the expected products (dopamine-sulfate, octopamine-sulfate) at acidic pH, we used cellulose thin-layer chromatography under strongly basic conditions for the separation. As shown in Figure 6 and quantified in Table 1, *p-*nitrophenol, dopamine, and *R,S*-octopamine were substrates for both *Ixosc *Sult 1 (R) and Sult 2 (R). The enzyme activity of expressed *Ixosc *Sult 1 (R) was found to be 1.3-1.5 nmol min^-1 ^mg^-1 ^and *Ixosc *Sult 2 (R) to be 0.3-0.4 nmol min^-1 ^mg^-1^, respectively, when incubated with 10 μM of each potential substrate. Product formation in the absence of enzyme (PAP^35^S plus substrate) was found to be zero. 17β-estradiol and pregnenolone showed no product formation under the conditions tested (data not shown). Because the ^35^S-labeled products of dopamine, octopamine, and *p-*nitrophenol migrated with a similar retention factor (R_*f*_), their individual formation was confirmed by mass spectral analysis. The sulfated product of dopamine and octopamine, respectively, were positively identified by mass spectral detection under negative ionization conditions ([dopamine-SO_3 _- H^+ ^+ Na^+^], 255.2 *m/z*; [octopamine-SO_3 _- H^+ ^+ Na^+^], 255.2 *m/z*) (Figure 7 A, B, C, D, E, F). *p-*Nitrophenylsulfate ion was detected in parallel incubations with both *Ixosc *Sult 1 and 2 (R) ([*p-*nitrophenol-SO_3 _- H^+^], 217.9 *m/z] *Additional file 1. Kinetics of the native blacklegged tick sulfotransferases for dopamine and octopamine were recently published separately [26].

**Table 1:** Quantitation of *Ixosc *Sult 1 (R) and 2 (R) activity.^a^

Reaction mixture	**Rate of reaction (nmol min**^**-1 **^**mg**^**-1**^)
PAP^35^S + Sult 1 (R) (Control)	0.00
*p-*Nitrophenol + Sult 1 (R) + PAP^35^S	1.30 ± 0.01
Dopamine + Sult 1(R) + PAP^35^S	1.52 ± 0.01
Octopamine + Sult 1(R) + PAP^35^S	1.34 ± 0.01
PAP^35^S + Sult 2 (R) (Control)	0.00
*p-*Nitrophenol + Sult 2 (R) + PAP^35^S	0.43 ± 0.01
Dopamine + Sult 2 (R) + PAP^35^S	0.39 ± 0.01
Octopamine + Sult 2 (R) + PAP^35^S	0.35 ± 0.01

^a ^Substrates (10 μM) were incubated with PAP^35^S (4 μM) and purified *Ixosc *Sult 1 (R)(0.04 mg/mL) or *Ixosc *Sult 2 (R)(0.03 mg/mL) for 30 min. Product formation in the absence of enzyme (PAP^35^S + substrate) was found to be zero.

## Discussion

In this study, we describe the expression of novel *Ixodes scapularis *sulfotransferase genes throughout the blacklegged tick life cycle and different states of blood feeding, and characterize their expressed recombinant proteins. *Ixosc *Sult 1 and Sult 2 are expressed during every life stage of blacklegged ticks, and interestingly, mRNA and protein levels are moderately but significantly changed (1.5 - 2.6 fold) upon blood-feeding at the nymphal and larval stages. We find it especially interesting that the process of feeding has an opposite effect on relative expression of the *Ixosc *Sult 1 and Sult 2 during both the larval and nymphal life stages, although no change in expression levels could be detected in the adult stage salivary gland or midgut (Figures 2, 3, 4). Thus, these two proteins seem to have different roles in the tick salivation and feeding process. *Ixosc *SULT 1 mRNA and protein were increased upon feeding from a low basal level at the larval and nymphal stage. In contrast, *Ixosc *SULT 2 mRNA and protein were decreased upon feeding from a high basal level at the larval and nymphal stage. Quantitative RT-PCR and quantification of band intensities by Kodak Digital Analyser confirmed the statistical increase of *SULT 1 *and decrease of *SULT 2 *as well as their gene products during feeding. Our recent publication [26] on homology modelling, molecular docking and enzyme kinetic studies of sulfotransferase activity from native tick tissues showed similar findings using specific tick homogenates. It should be noted that these results were obtained from three pools of tissue derived from multiple ticks, and that the blood-fed ticks were sampled after being attached to hosts for 48 - 72 hours. Because tick salivation and secretion is a dynamic process throughout the multi-day feeding, experiments are in progress to profile the expression during more exact time periods. While there is evidence for altered expression of multiple genes during tick feeding [31], surprisingly little is known about the mechanism of gene expression in tick salivary glands. Furthermore, the exact tissue localization of the sulfotransferases in the nymphal and larval stage has not yet been determined; whole homogenates were used of unfed and blood-fed ticks at these life stages.

The related sulfotransferase (ceST) isolated from *C. elegans *catalyzed sulfonation of a variety of phenols at the rate of 0.1-0.5 nmol min^-1 ^mg^-1^, but no endogenous substrate was identified [10]. The sulfotransferase (bmST) isolated from *Bombyx mori *was found to catalyze sulfonation of 4-nitrocatechol at 0.2 nmol min^-1 ^mg^-1^, although no endogenous substrate was identified [11]. The cytosolic sulfotransferases from *D. melanogaster *catalyzed sulfonation of vanillin, 1-naphthol, *p-*nitrophenol, ecdysone, and dopamine [13] at rates of 0.2-1 μmol min^-1 ^mg^-1^. The published rates of sulfonation by bmST and ceST are similar to those measured for *Ixosc *Sult 1 and 2.

The well-characterized human sulfotransferases catalyze sulfonation of a variety of endogenous hormones and neurotransmitters, as well as dietary phenols and environmental contaminants. In general, the sulfotransferases responsible for sulfonating endogenous substrates have high affinity for these substrates. Human SULT1A3, which is also called dopamine sulfotransferase, has a Km of 4 μM for dopamine, while human SULT1A, generally called phenol sulfotransferase, has a Km of 130 μM for dopamine. Human SULT1E1, which is also called estrogen sulfotransferase, has a Km of 1-20 nM for 17β-estradiol, while human SULT1A1 and SULT2A1 can sulfonate 17β-estradiol with Km of 1-30 μM. In our earlier findings, dopamine was found to be a good substrate (Km of 0.1-0.4 μM) for the native tick sulfotransferases irrespective of their feeding stage where as octopamine served as a substrate only after feeding [26].

Interestingly, in this present study, we found that both dopamine and *R,S-*octopamine could serve as substrates for purified *Ixosc *Sult 1 (R) and Sult 2 (R) at a concentration of 10 μM. We chose 10 μM as a test concentration because of our detection method's limit of sensitivity. Further studies are needed to explore the physiological relevance of *Ixosc *Sult 1 and Sult 2 sulfonation of tick neuroeffectors dopamine and octopamine, and to identify possible additional sulfotransferases in *Ixodes scapularis*.

We find the sulfonation of dopamine and octopamine to be noteworthy because salivary secretion in feeding ticks is under neuronal control. Tick salivary gland secretion is stimulated by dopamine in the neuroeffector junction via dopamine D1 receptor activation of adenylate cyclase and an increase in intracellular cAMP [27,32]. Dopamine also opens a voltage-gated Ca^2+ ^channel allowing an influx of extracellular calcium that stimulates a cytosolic phospholipase A2, effecting release of sequestered arachidonic acid in tick salivary glands that is subsequently converted to prostaglandins [33]. Prostaglandins are secreted at extremely high (μM) levels into tick saliva for export to the host [27] where they exhibit anti-hemostatic, vasodilatory, immuno-suppressive, and anti-inflammatory activities [34-36]. Thus, dopamine could modulate salivation and/or prostaglandin production in the tick salivary gland. Octopamine acts as a neuromodulator in arthropods with a similar mechanism to dopamine [30]. In insects, octopaminergic neurons modulate a variety of target tissues, which include skeletal muscles, heart and oviduct [37]. However, the exact tissue localization of the sulfotransferases in nymphal and larval blacklegged ticks has not yet been determined; whole homogenates were used of unfed and blood-fed ticks at these life stages.

In contrast to vertebrates, invertebrates use a variety of enzymatic routes to metabolise monoamines. These routes include sulfonation, N-acetylation, gamma-glutamyl conjugation, sugar conjugation, β-alanyl conjugation, as well as oxidative deamination [38]. Catabolism is one of the effective mechanisms for dopamine inactivation. In humans, this involves multiple pathways that include oxidative deamination by monoamine oxidase (MAO), O-methylation by catechol-O-methyltransferase (COMT) and conjugation by sulfotransferase [39]. Regarding ticks, reports from Atkinson *et al. *[40] confirm the presence of monoamine oxidase (MAO) in the homogenates of *B. microplus *larvae, and MAO inhibition potentiated the dopamine effect on fluid secretion from salivary glands of *A. hebraeum in vitro *[41,42]. According to *in vivo *experiments by Kaufman and Sloley [42], it is clear that MAO is not the only means for disposing of biogenic amines in ticks. Octopamine *N*-acetyltransferase (with a Km for octopamine of 4 μM) has been demonstrated in the synganglion of *B. microplus *[43]. Thus, multiple pathways may be available for inactivating monoamines in tick species. Indeed, despite the recent advances in tick neurobiology and the identification of key genes, tick neuroscience lags behind that other invertebrates [44].

*Ixosc *Sult 1 or Sult 2 may serve as critical modulators of the prostaglandin synthesis pathway or as modulators of salivary secretion. Because all neuronally released biogenic amines have relatively short half-lives to prevent their action from continuing after the neuronal signal arrests, we thus propose that the two tick sulfotransferases described herein could function by metabolically altering the biological signal for salivary secretion in *I. scapularis *even though both of these genes were expressed differently. We are currently following up on these studies to explore their physiological relevance to tick feeding.

## Conclusions

The salivary glands of ticks, in addition to their role in feeding, serve a role in ion and water metabolism. In a blood-feeding tick, production of saliva is the main mechanism of water excretion, ie. ticks alternate blood ingestion and salivation. Therefore, interrupted or inappropriate sulfation of neurotransmitter molecules involved in salivation could negatively affect a tick's ability to alternate the cyclic salivation and blood sucking process, potentially inhibiting blood feeding and pathogen transmission. In this study, we observed that expression of *Ixosc *Sult 1 is up-regulated, while expression of *Ixosc *Sult 2 is down-regulated during blood feeding at both the larval and nymphal stages, suggesting that these sulfotransferases have different functions in the salivation/feeding process of blacklegged ticks, even though expressed recombinant proteins sulfonate both dopamine and octopamine. Further experiments, perhaps using sulfotransferase gene knockdown in these ticks may help clarify the significance of our observations.

## Methods

### Reagents

Restriction enzymes, Taq DNA polymerase, Plasmid DNA and polymerase chain reaction (PCR) product purification kits, Block-iT T7 TOPO linker were purchased from Invitrogen (Carlsbad, CA, USA) and Qiagen (Valencia, CA, USA). Mouse IgG-HRP was purchased from StressGen (Victoria, BC, Canada). The prokaryotic expression vector pTrc His2 Topo TA was purchased from Invitrogen, CA, USA. Enzyme chemiluminescent detection system was obtained from KPL laboratories. [^35^S]-3'-phosphoadenosine-5'-phosphosulfate (PAP^35^S) was purchased from Perkin Elmer Life and Analytical Sciences (1.1 - 2.43 Ci/mmol, solution in 1:1 ethanol: water). Unlabeled PAPS was purchased from Sigma Chemical and was purified before use by HPLC separation.

### Amino acid sequence comparisons

The *Ixosc *Sult 1 and Sult 2 sequences were compared with a set of known cytosolic sulfotransferase family. Multiple sequence comparisons were conducted using Accelrys SeqLab (GCG version 11.1). Human sulfotransferases are listed with a protein data bank code for a matching x-ray crystal structure. *C. elegans *and *B. mori *sulfotransferases are listed with their UniProt code.

### Tick collection and feeding

*Ixodes scapularis *ticks collected as unfed adults from forests in southern Rhode Island were allowed to blood feed on New Zealand white rabbits under controlled laboratory conditions [45]. A restraining collar was placed around the neck of each rabbit, and their ears were covered with cotton socks prior to tick exposure. These engorged adult ticks laid eggs, and hatched larvae were reared in the laboratory by blood feeding on golden hamsters to produce nymphs. To produce partially engorged experimental ticks, larvae and nymphs were allowed to feed on mice and adult ticks fed on New Zealand white rabbits [45]. The length of feeding of the larvae and nymphs was 48-72 hr, and the feeding time of the adults was 72 hr. All animal studies were approved by the Institutional Animal Care and Use Committee (protocol number AN01-12-014).

### Tick tissue samples

Three pooled samples from eggs, larvae and nymphs (~30 - 50) were homogenized and used for the studies. Three tissue pools from salivary gland or midguts were dissected from adult ticks (~30 adults). Adult tick salivary gland or midgut tissue was dissected in ice-cold 100 mM 3-(N-morpholino)-propanesulfonic acid (MOPS) buffer containing 20 mM EGTA, pH 6.8. When used for isolating total RNA, tissues were washed gently in MOPS/EGTA buffer and immediately stored in RNA later. For Western blotting, tick tissues were used immediately after being dissected or were stored at -70°C in 0.5 M piperazine-N,N-bis-2-ethane sulfonic acid, pH 6.8, containing 20 mM EGTA, 1X Complete Mini-Protease™ Inhibitor Cocktail (Roche) and 40% glycerol (v/v). All manipulations were carried out at 4°C.

### Quantitative Expression of *Ixosc *SULT 1 and SULT 2 genes-qRT-PCR

Gene specific primers [GenBank: DQ066225.1 and GenBank: DQ066226.1] of *Ixosc SULT 1 *and *SULT 2 *genes [5] were designed. Total RNA was isolated using an RNAqueous^@ ^total RNA isolation kit (Ambion) from eggs, unfed larvae, unfed nymphs, unfed adults, 48-72 hr fed larvae, 48-72 hr fed nymphs, and 72 hr fed adults. Concentration and purity of total RNA was determined spectrophotometrically at 260 and 280 nm, aliquoted and stored at -80°C until use.

Real-time quantitative PCR was performed using the Mx4000 or Mx3005P Multiplex Quantitative PCR system and the Brilliant SYBR Green Single Step QRT-PCR Master Mix Kit (Stratagene, La Jolla, CA) according to the manufacturer's instructions. A standard curve of 10^0^-10^7 ^copies per reaction were generated using purified *Ixosc SULT 1 *and *SULT 2 *PCR products as the template. The following primers were used for all reactions: *SULT 1 *Forward 5'ACATGATCTGGGGCGACTAC3' and Reverse 5'GTCAAGGGTGCTCTCGTCTC3', *SULT 2 *Forward 5'GTTATGCTCTGCGACACGAA3' and Reverse 5'ACTCACGTCGAACGTCCTCT3'. Reactions contained 10 ng of RNA were run under the following conditions: 1 cycle of 50°C for 30 min and 90°C for 15 min followed by 40 cycles of 95°C for 30 s and 51°C for 30 s. Fluorescence was measured every cycle at the end of the 51°C step. The copy number of *SULT 1 *and *SULT 2 *mRNA in each sample was determined using the Mx4000 or Mx3005P data analysis software based on the standard curve. The relative quantity of target mRNA in each sample was normalized to β-actin mRNA. All the reactions were done in triplicate.

### Cloning, sequencing and expression of *Ixosc *SULT 1 and *Ixosc *SULT 2 in *E. Coli*

After PCR amplified full-length *Ixosc SULT 1 *and *Ixosc SULT 2*, gel-purified fragments were cloned into the pTrcHis2 TOPO TA expression vector. Standardized methods were followed to insert plasmid into Top10 *E. coli *(Invitrogen), and the proper insertion was confirmed. The pTrcHis2 TOPO TA expression vector contains an ampicillin resistance gene, IPTG promoter, and six-residue histidine tag. Expression was induced with IPTG treatment (0.3 mM) for 3 hrs. Cells were treated with 0.3 mg/mL lysozyme for 30 min, washed twice with homogenization buffer, and homogenized by sonication. Homogenization buffer consisted of 20 mM potassium phosphate pH 7.0 with 1 mM dithiothreitol, and 1 mM PMSF. Cytosolic fractions were isolated by preparation of 100,000 × *g *supernatant. For purification of expressed *Ixosc *Sult 1 and *Ixosc *Sult 2, cytosol was loaded onto a HIS-Select ILAP (Sigma) immobilized metal affinity chromatography resin, and eluted with 50 mM imidazole and 500 mM NaCl. Eluted protein was passed through the desalting column (Zeba Desalt Spin columns, Pierce, USA) to remove imidazole and sodium chloride before being used for enzyme assays. Protein purity was assessed using denaturing gel electrophoresis with Coomassie blue staining or Western blot detection. Hereafter the purified recombinant proteins were referred as *Ixosc *Sult 1 (R) and Sult 2 (R). The expressed enzyme was detected using Anti-His (C-term) antibody (Invitrogen, USA).

### Polyclonal antibody to expressed purified *Ixosc *Sult 1 (R) or Sult 2 (R)

Expressed and purified *Ixosc *Sult 1 (R) and Sult 2 (R) were used separately to immunize BALB/c female mice (4 to 6 weeks old, Charles River, three mice per each purified protein). Mice were injected intra muscularly four times over six weeks (at two week intervals) with 20 μg of purified protein in each treatment. Antibody production was assessed by sampling venous blood and using it as probe for Western blots.

### Western blotting

Pooled salivary glands, midgut or whole tissue (3 different pooled samples) from each tick life stage (unfed larvae, unfed nymph, unfed adult, 48-72 hr fed larvae, 48-72 hr fed nymph, and 72 hr fed adult) were homogenized (1 min sonication) in ice-cold extraction buffer (PBS, 1 mM dithiothreitol, 2.5 mM EGTA, and 1X Complete Mini-Protease™ Inhibitor Cocktail (Roche)). Additionally, affinity-purified *Ixosc *Sult 1 (R) and Sult 2 (R) were used as positive controls. Protein concentration was estimated by the Bradford method [46]. Tick salivary gland protein extracts (20-30 μg each life stage or tissue) were separated by 10% SDS-polyacrylamide gel electrophoresis (SDS-PAGE) [47] and then transferred onto nitrocellulose membranes in a Transblot cell (Bio-Rad) following the manufacturer's instructions [48]. Nonspecific protein binding sites were blocked with 5% skim milk, and the membranes were incubated with polyclonal *Ixosc *Sult 1 (R) or *Ixosc *Sult 2 (R) antibodies at a dilution of 1:2000. Antigen-antibody complexes were visualized with horseradish peroxidase-conjugated anti-mouse IgG (StressGen) at a dilution of 1:10,000 and detected with SuperSignal chemiluminescent peroxidase substrate (Pierce, Rockford, IL, USA) on a Bio-Rad ChemiDoc XRS imaging system. All reactions were done in triplicates. The net intensity/band intensity was quantified using Kodak digital 1D image analysis software.

### Sulfotransferase activity

Ability of expressed *Ixosc *Sult 1(R) and Sult 2(R) to catalyze sulfonation of test chemicals was assessed using expressed purified enzyme preparations (in 20 mM potassium phosphate pH 7.0) and PAP^35^S (in water). For these assays, the ethanol: water solvent of the commercial PAP^35^S was evaporated in vacuum and the residue reconstituted in water. Potential substrates (dopamine, *R,S*-octopamine, *p*-nitrophenol, 17β-estradiol and pregnenolone) were dissolved in water and diluted into the incubation mixture to the final concentration of 10 μM. Assays generally contained 0.03-0.08 mg/mL purified expressed protein and 0.01 mCi/mL (4-9 μM) PAP^35^S. After 30 min at 37°C, reactions were stopped by heat inactivation and pelleting of denatured protein. Sulfated metabolites were identified by mass spectrometry as described below. For quantification, supernates were spotted onto the cellulose thin-layer chromatographic (TLC) paper, and components were separated by capillary movement of the mobile phase (2-propanol: ammonium hydroxide: water, 6:3:1) up the paper [49]. Radiolabeled components were visualized and quantified after phosphor transfer (Kodak TR storage phosphor screen #9715) with subsequent phosphor imaging on a Typhoon 9410. All experiments were done with at least three replications.

The sulfated metabolites were identified by mass spectrometry on a QSTAR^® ^Elite LC/MS/MS-TOF (Applied Biosystems, USA) in the negative ion mode. Incubation supernates were directly injected through a syringe filter (0.45 μm) and ionized by turbo-electrospray. Ions were scanned over a region of 50-1000 m/z. Background scans were collected just before injection of sample. All experiments were done at least in triplicates.

### Statistical Analysis

Where indicated, values were expressed as mean ± SD of three determinations. Statistical differences were carried out by Sigma Stat version 3.5 (Systat Software Inc. San Jose, CA) student's t-test. p values of < 0.05 were considered statistically significant.

## Authors' contributions

SP carried out the molecular genetic studies, design and executed the experiments and drafted the manuscript. EBY carried out the sulfotransferase assays and analyses. JMCR was involved in part of the experimental design and in critically revising the final manuscript. RSK was involved in the experimental design, experiment coordination, and in critically revising the manuscript. TNM was involved in the experimental design, experiment coordination, and in critically revising the manuscript. All authors have approved the final manuscript.

## Supplementary Material

Additional file 1 **Mass Spectra of p-nitrophenyl sulfate**. Mass Spectra of p-nitrophenyl sulfate formed by incubating *Ixosc *Sult 1(R) or *Ixosc *Sult 2 (R). a) Sult 1(R) + PAP^35^S + p-nitrophenol, b) Sult 2 (R) + PAP^35^S + p-nitrophenol. Click here for file
