# Supplementary figures and images for: Molecular characterization of novel sulfotransferases from the tick, Ixodes scapularis
Source: BMC Biochem. 2011 Jun 27;12:32. doi: 10.1186/1471-2091-12-32 (PMC3150262; doi:10.1186/1471-2091-12-32)

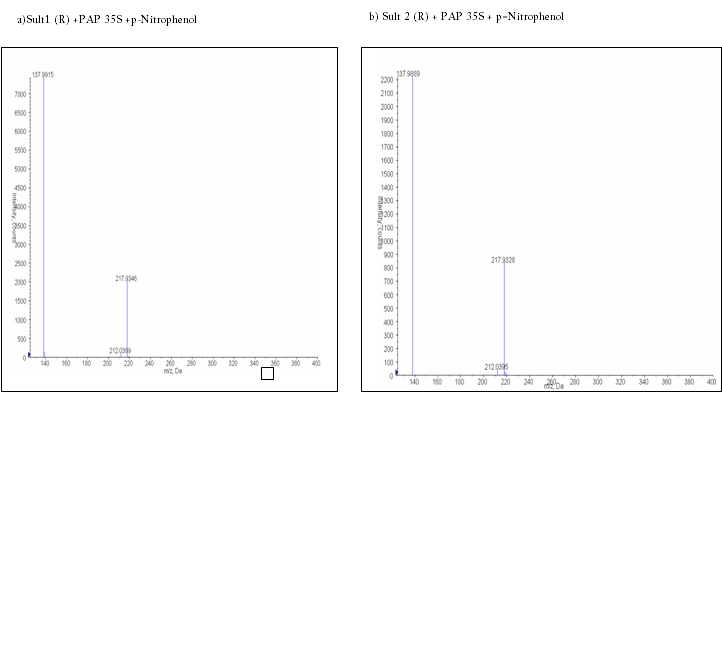

Supplement: Additional file 1 — Mass Spectra of p-nitrophenyl sulfate. Mass Spectra of p-nitrophenyl sulfate formed by incubating Ixosc Sult 1(R) or Ixosc Sult 2 (R). a) Sult 1(R) + PAP35S + p-nitrophenol, b) Sult 2 (R) + PAP35S + p-nitrophenol. [file 1471-2091-12-32-S1.PNG]
